# Economic burden of sports injury in China: result from a single center of medical quality and safety monitor system

**DOI:** 10.1186/s12962-025-00659-z

**Published:** 2025-12-02

**Authors:** Tsz-ngai Mok, Zhiguang Huang, Ning Ruoyu, Jian Guan, Jing Zhao, Zihang Chen, Lek-Hang Cheang, Man-seng Tam, Dongyi Fan, Tien-cheng Yeh, Sicun Li, Huajun Wang, Xiaofei Zheng, Wai-kit Ming

**Affiliations:** 1 Department of Infectious Diseases and Public Health, City University of Hong Kong, Hong Kong SAR, China; 2 Department of Orthopedics, The Third Hospital of Shijiazhuang, Shijiazhuang, China; 3 State Key Laboratory of Quality Research in Chinese Medicine, Department of Pharmaceutical Sciences, Faculty of Health Sciences, Institute of Chinese Medical Sciences, University of Macau, Macau, China; 4 Department of Sports Medicine, The First Affiliated Hospital, Guangdong Provincial Key Laboratory of Speed Capability, The Guangzhou Key Laboratory of Precision Orthopedics and Regenerative Medicine, Jinan University, Guangzhou, China; 5 Department of Psychology, Li Ka Shing Faculty of Medicine, State Key Laboratory of Brain and Cognitive Sciences, The University of Hong Kong, Hong Kong SAR, China; 6 Department of Orthopedic Surgery, Centro Hospitalar Conde de Sao Januario, Macau, China; 7 IAN WO Medical Center, Macao Special Administrative Region, Macau, People’s Republic Of China; 8 Institute of Global Governance and Innovation for a Shared Future, The City University of Hong Kong, Hong Kong, Hong Kong S.A.R., China; 9 Department of Family Medicine and Primary Health Care, Kowloon Central Cluster, Hospital Authority, Kowloon, Hong Kong S.A.R., China

## Abstract

**Background:**

Sports injuries are becoming increasingly prevalent worldwide as sports and physical activities gain popularity. These injuries impose a significant burden on individuals and society. However, a limited understanding of the cost analysis of sports injuries in Southeast China exists.

**Objective:**

The objective is to explore the medical costs associated with sports injury surgery and related healthcare policies from a hospital perspective.

**Methods:**

We conducted a prospective analysis of inpatient costs for sports injury surgeries at the First Affiliated Hospital of Jinan University from 2015 to 2022. The total expenditure was categorized into various components: general medical service, nursing service, imaging, rehabilitation, etc. These were analyzed based on the International Classification of Function, Disability, and Health (ICF) system, surgical type, year category, and age. Furthermore, we accessed authoritative economic data, such as standardized occupational salaries and cost estimations, to comprehensively depict the economic burden. We employed a generalized linear model to identify factors influencing costs and performed statistical comparisons across different demographic and clinical categories.

**Findings:**

All values are expressed in 2022 Chinese Yuan (¥). A total of 8,804 hospitalized patients were registered. The median total expenditure is ¥38,691 (19680, 49640) (≈ 5749 US dollars), of which the S730 is the lowest ¥23,250 (8627, 31364) (≈ 3455 USD), and the S710 is the highest ¥61,626 (25990, 77122) (≈ 9157 USD). Grouped by year, the median in 2019–2020 was the highest at ¥42,008 (23095, 53281) (≈ 6242 USD), and the lowest in 2015–2016 was ¥30,903 (11517, 42954) (≈ 4592 USD). In terms of surgery type, Shoulder Arthroscopy has the highest median cost of ¥51,550 (40703, 60028) (≈ 7660 USD), and Ankle arthroscopy has the lowest median cost of ¥34,177 (29194, 38209) (≈ 5078 USD). In terms of age, the highest median cost was ¥44,306 (27807, 55588) (≈ 6583 USD) for > 65 years, and the lowest was ¥13,671 (5182, 15899) (≈ 2031 USD) for 1–12 years. Different age groups, genders, years of admission, ICF categories, surgical grades, occupation types, and arthroscopic surgery types significantly affect hospitalization costs.

**Conclusion:**

This study examined various aspects of surgery for sports injuries in Southeast China. Shoulder arthroscopy was the costliest procedure, with disposable medical materials being the primary expense. These findings offer valuable insights into expenditure patterns in this context.

**Clinical trial number:**

Not applicable.

**Supplementary Information:**

The online version contains supplementary material available at 10.1186/s12962-025-00659-z.

## Introduction

Sports injuries are a global health problem that burdens individuals and society greatly [1–3]. Sports injuries have increased in recent years, affecting people of all ages [4]. At the same time, they also place a substantial financial burden on the healthcare system, but there is a notable absence of comprehensive research on their economic impact.

Sports injuries such as fractures, sprains, strains, and other injuries related to muscles, ligaments, or joints can significantly affect an individual’s quality of life and productivity [5]. Considering the significant impact of sports injuries on individuals and society [6], it becomes imperative to understand their economic burden comprehensively. This understanding can facilitate the efficient allocation of resources, implementation of preventive measures, and improvement of sports medicine treatments. Despite the plethora of studies investigating the economic burden of sports injuries globally, our current knowledge regarding the economic impact of various sports injuries remains limited. A systematic evaluation by Bielska et al. [7] analyzed health economics papers on ankle-foot sprains and fractures worldwide from 1980 to 2014. The review found that differences in injury type and study characteristics led to different treatment costs across studies, which affected the overall findings. Although direct comparisons of treatment costs are possible, the review also showed that expenses incurred by patients and healthcare systems tend to increase with the complexity of the injury. However, our understanding of the health economics of sports injuries in southeastern China remains inadequate. The personal and socioeconomic impact of sports injuries can be revealed by analyzing data on medical costs, rehabilitation costs, lost income, and other relevant factors. Such a comprehensive survey could provide decision-makers solid evidence to develop effective strategies and policies. Understanding the true impact of sports injuries will enable us to allocate resources more effectively, implement preventive measures, and improve treatments, thereby improving the overall management of sports-related health problems.

From a hospital perspective, this study aimed to describe the current sports injuries in southeastern China using data from a single-center database and to analyze the economic burden of sports injuries. The study collects and analyzes various sports injury data, such as patient demographics, injury type, treatment methods, and cost information. By analyzing this data in depth, we expect to draw practical conclusions about the economic impact of sports injuries. Our findings will provide valuable insights for policymakers, healthcare providers, and researchers to make informed decisions and develop effective strategies to address the challenges of sports injuries.

## Methods

### Design

A flowchart of the study is presented in Supplementary Fig. S1. The Consolidated Health Economic Evaluation Reporting Standards (CHEERS) checklist [8] was adopted to guide and qualify the study (Supplementary Table S1).

### Data sources

All data in this study are sourced from the First Affiliated Hospital of Jinan University. All patient information is entered into the Chinese Medical Quality And Safety Monitor System. The surgical records include gender, race, date of birth, date of hospitalization, date of operation, date of discharge, past information such as medical history, current health status, outpatient and surgical diagnoses, surgical notes, blood transfusion records, complications, discharge status, and total hospital charges. Standardized diagnoses were made using the International Classification of Functioning, Disability, and Health (ICF) to assess individuals for impairments in functioning and limitations in participation. The ICF classification system provides a comprehensive framework for describing and understanding the impact of a disease or health problem on individual functioning, activity, and participation. The ICF classification system can better assess an individual’s functioning level and participation in daily life and social activities [9]. Occupational salaries were standardized through the Southeast China Statistical Yearbook for subsequent estimation of income loss.

### Enrollment

All patients who underwent surgery for sports injuries at the hospital from 2015 to 2022 were identified, and 28,381 records were initially retrieved. The study was approved by the ethics committee of Jinan University (approval number: KY-2023-220) in accordance with the Declaration of Helsinki. After further examination of these records, cases that were not included in the study, incomplete or duplicated, were excluded. Eight thousand eight hundred-four patients were included in the subsequent analysis (Supplementary Fig. S1). Baseline features are retrieved from the database. These factors include age, sex, occupation, source of patient, name of the disease and detailed expenditure (total expenditure, income loss, general medical service, general treatment operation, nursing service, pathological diagnosis, other, laboratory examination, imaging, clinical diagnosis, non-surgical treatment, surgical treatment, rehabilitation, total cost of medicine, blood transfusion, consumable medical items for examination, disposable medical materials, travel expenses).

### Planned analyses

In the economic analysis, each patient will be counted once. First, due to the complexity of sports injury surgery, the ICF was used to stratify the risk of each surgery. S710-S760 stands for head and neck respectively region, shoulder region, upper extremity, pelvic region, lower extremity, and trunk. The medical costs for each ICF category were also assessed. Second, each year’s total number of sports injury surgeries was aggregated to explore the annual differences in medical expenditure. Third, subgroup analyses were performed by type of surgery, including shoulder arthroscopy, knee arthroscopy, ankle arthroscopy, and others. Shoulder arthroscopic surgery treats shoulder-related problems such as rotator cuff tears, acromion impingement syndrome, and shoulder dislocations. Knee arthroscopic surgery mainly treats knee-related problems such as meniscus injuries, ACL tears, and cartilage wear. Ankle arthroscopic surgery mainly treats ankle-related problems such as synovitis, cartilage damage, and instability [10]. Fourth, five age groups were distinguished to investigate differences in expenditures: 1–12 years, 13–18 years, 19–45 years, 46–65 years, and >65 years. Fifth, according to the characteristics of sports injuries and clinical practice, Different age groups, genders, years of admission, ICF categories, surgical grades, occupation types, and arthroscopic surgery types were considered potential factors affecting the cost of sports injuries and included in the regression model.

### Direct medical cost

Direct medical cost data were collected by accessing hospital records and health insurance data. We extracted medical cost information from patients who participated in the study for a specific period. Cost calculations were based on medical bills and standardized fee schedules, such as General medical service and nursing service. We calculated the mean cost and the upper and lower quartiles of the cost using descriptive statistics due to the heavily skewed values. All data processing and analyses were performed to protect patient privacy, and anonymous coding measures were taken.

### Indirect cost

According to the type of occupation in personal information, the Southeast China Statistical Yearbook normalizes professionals’ salaries. We use the age to infer the working age to get the amount of annual leave and combine the hospitalization time to calculate the income loss. After Chinese laborers have worked in the unit for one year, ten years, and twenty years, their annual paid leave days are five days, ten days, and fifteen days, respectively. The number of vacation days per year is five days.

$$(Income loss = (hospital days-annual leave) * occupational daily salary)$$

At the same time, we calculate the fare range according to the distance according to the classification of the source of the patient in the patient information. To be more realistic, each patient’s occupational daily salary and fare are randomly generated within the estimated range of the corresponding category.

### Statistical analysis

All cost values are expressed in 2022 Chinese Yuan (Yuan) and adjusted as necessary. Statistical analyses were performed using SPSS Statistics Version 23.0 (IBM, Armonk, NY, USA) and Python (Version 3.1.1). Categorical variables (diagnosis and baseline demographic characteristics) are presented as frequencies (percentages). Continuous variables (baseline demographics and cost data) were presented as medians (lower quartile, upper quartile) and tested with nonparametric tests (Kruskal-Wallis test). A generalized linear model (GLM) with a log link and gamma distribution was adopted to identify the factors contributing to total expenditures. Expenditures are compared across statistical categories, years, and ages. A *P* value < 0.05 indicates a significant difference between the two groups.

## Results

### Baseline demographic characteristics

The study identified and analyzed 8,804 hospitalized patients undergoing surgery for sports injuries from an initial search of 28,381 records. The mean age of these patients was 46.81 ± 20.49 years. There were 4,992 male patients and 3,812 female patients.

In terms of occupations, there are 330 professional and technical personnel, 155 self-employed people, 120 civil servants, seven military personnel, 476 farmers, one medical staff, 876 students, nine educators, 58 unemployed people, one service industry employee, 23 managers, 1,086 employees, three transportation workers, and 1,240 retirees.

Regarding the sources of patients, there were 2999 patients from the district or city where the hospital is located, 2182 patients from other districts (counties) of the city where the hospital is located, five foreign patients, four patients from other provinces and cities, and four patients from other provinces (municipalities). There are 1,514 patients from Southeast China, 15 from other districts and counties in this city, 2,067 from other towns in this province, and 18 from Hong Kong, Macao, and Taiwan.

According to the ICF classification, there were 57 patients with S710 injury, 987 patients with S720 injury, 2216 patients with S730 injury, 24 patients with S740 injury, 4496 patients with S750 injury, and 1024 patients with S760 injury. Table 1 provides details on baseline characteristics.

### Expenditure according to the ICF category

**Fig. 1:**
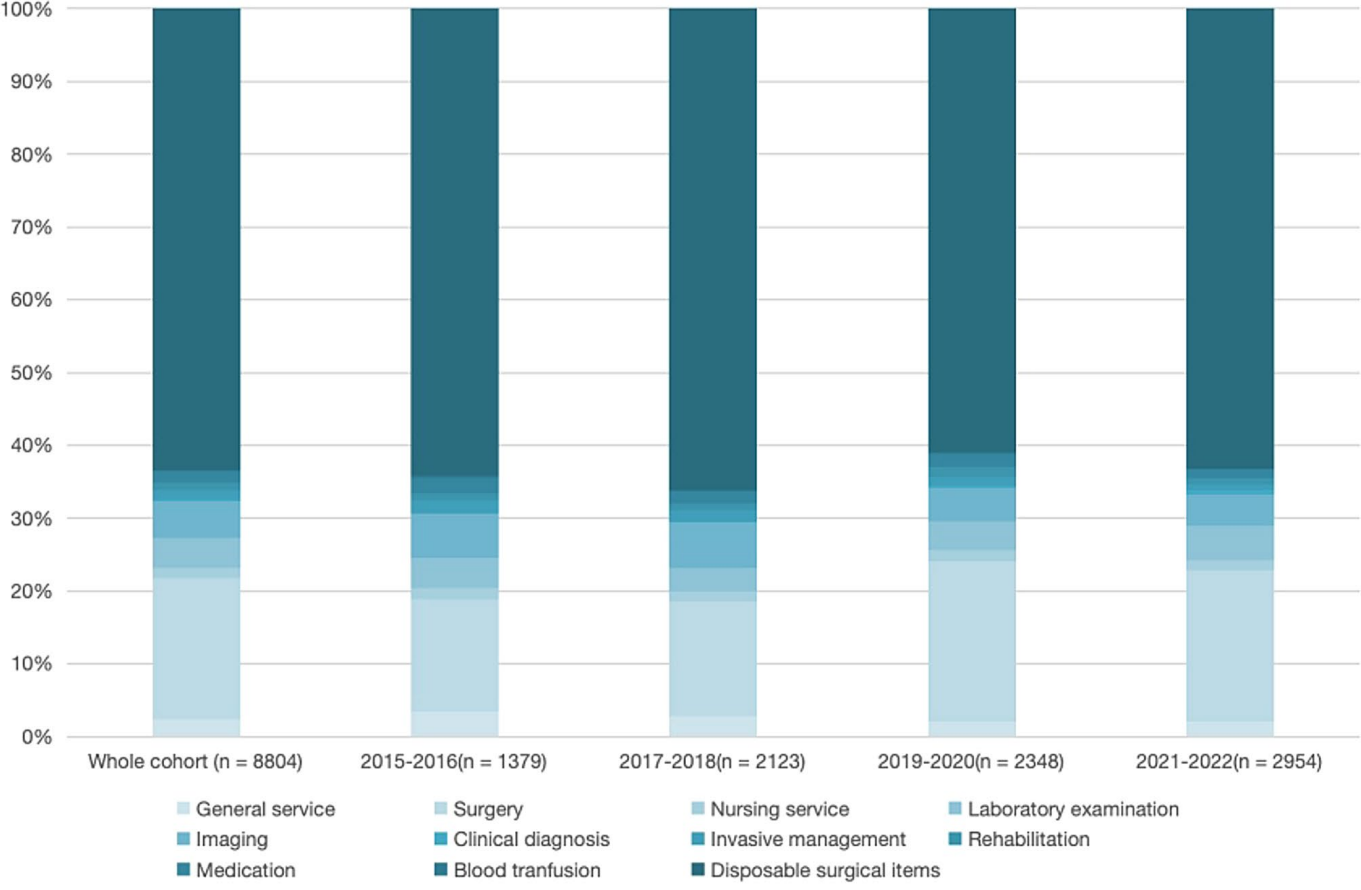
Proportion of each subject in the total expenditure according to year category

The median total cost was 38,691 yuan (≈ 5749 USD), and there were significant differences among the six statistical categories (*P* < 0.001). Among them, s730 was the lowest (23,250 yuan ≈ 3455 USD), and s710 was the highest (61,626 yuan ≈ 9156 USD). The cost structure is based on the cost of disposable medical materials for surgery (26,779 yuan ≈ 3979 USD), surgical treatment (8,748 yuan ≈ 1300 USD), Imaging (3,905 yuan ≈ 580 USD) Mainly, the differences between different statistical categories are statistically significant (*P* < 0.001). The total costs of s720, s740, s750, and s760 are relatively close, much higher than s730 and lower than s710. It is worth noting that s720 is higher than other ICF classifications in surgical treatment fees and rehabilitation expenses (Fig. 1).

**Fig. 2:**
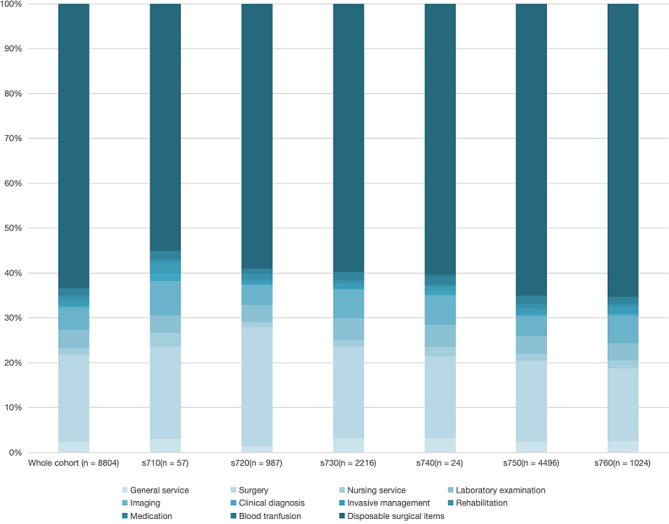
Proportion of each subject in the total expenditure according to ICF category

**Fig. 3:**
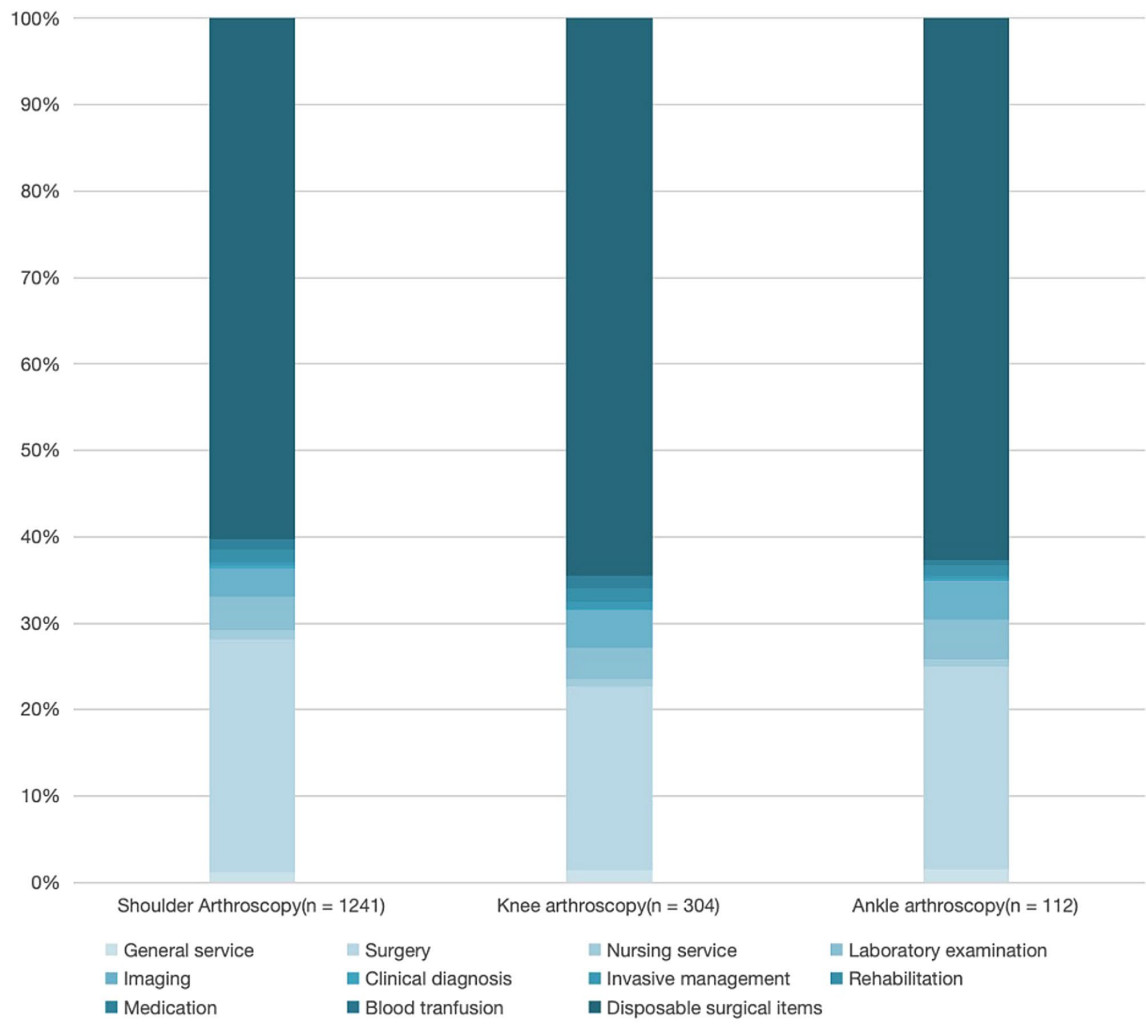
Proportion of each subject in the total expenditure according to arthroscopy category

**Fig. 4:**
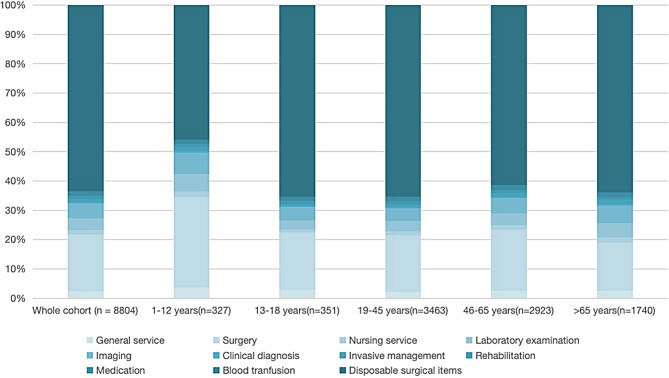
Proportion of each subject in the total expenditure according to age category

Detailed expenditure by statistical category is provided in Table 2; Figs. 2–4.

**Table 1:** Expenditure of patients according to ICF category

Expenditure (¥)	Whole cohort (*n* = 8804)	s710(*n* = 57)	s720(*n* = 987)	s730(*n* = 2216)	s740(*n* = 24)	s750(*n* = 4496)	s760(*n* = 1024)	*P* value
**Direct Cost**								
Total expenditure	38,691 (19680, 49640)	61,626 (25990, 77122)	45,733 (36906, 56609)	23,250 (8627, 31364)	40,977 (10747, 50368)	43,179 (25177, 52468)	44,281 (23746, 52364)	< 0.001*
General medical service	794 (300, 838)	1378 (715, 1615)	571 (303, 588)	589 (225, 675)	1120 (458, 1631)	904 (354, 940)	932 (375, 1058)	< 0.001*
General treatment operation	745 (195, 690)	1836 (681, 1986)	353 (196, 370)	566 (203, 643)	1141 (269, 1541)	860 (193, 767)	938 (192, 903)	< 0.001*
Nursing service	492 (158, 547)	1548 (415, 1938)	468 (299, 581)	273 (93, 324)	753 (198, 875)	556 (176, 588)	646 (169, 618)	< 0.001*
Pathological diagnosis	19 (0, 0)	24 (0, 0)	14 (0, 0)	12 (0, 0)	26 (0, 0)	21 (0, 0)	30 (0, 0)	< 0.001*
Other	20 (0, 0)	125 (0, 0)	4 (0, 0)	18 (0, 7)	30 (0, 12)	22 (0, 0)	28 (0, 28)	< 0.001*
Laboratory examination	1363 (761, 1509)	1927 (894, 2295)	1553 (874, 1884)	931 (561, 1074)	1701 (752, 2099)	1498 (840, 1662)	1483 (856, 1539)	< 0.001*
Imaging	1714 (682, 2361)	3905 (1746, 5415)	1839 (998, 2461)	1252 (433, 1730)	2358 (1049, 3027)	1739 (765, 2395)	2348 (1048, 3009)	< 0.001*
Clinical diagnosis	123 (0, 90)	924 (0, 512)	103 (0, 90)	59 (0, 36)	304 (0, 230)	136 (0, 90)	179 (0, 47)	< 0.001*
Non-surgical treatment	401 (36, 342)	1282 (188, 1291)	336 (122, 369)	215 (0, 147)	388 (50, 386)	449 (77, 385)	607 (0, 431)	< 0.001*
Non-surgical treatment, including clinical physical therapy	186 (0, 184)	378 (73, 453)	187 (92, 199)	101 (0, 37)	175 (0, 210)	207 (0, 214)	265 (0, 163)	< 0.001*
Surgical treatment	5809 (2907, 8073)	8748 (290, 12269)	10,567 (8557, 13339)	3425 (1960, 4092)	5312 (2374, 6723)	6033 (3494, 8008)	5249 (2373, 6560)	< 0.001*
Surgical treatment, including anesthesia	901 (95, 1045)	1825 (0, 3363)	2167 (730, 3477)	501 (0, 735)	1397 (24, 1073)	832 (287, 1054)	786 (0, 924)	< 0.001*
Surgical treatment, including surgical	4906 (2451, 7046)	6923 (7, 9870)	8400 (7166, 10563)	2916 (1786, 3420)	3915 (2315, 3572)	5200 (2941, 7123)	4463 (1977, 5079)	< 0.001*
Rehabilitation	347 (30, 428)	247 (20, 407)	605 (94, 941)	119 (20, 90)	111 (20, 82)	437 (31, 671)	203 (20, 153)	< 0.001*
Total cost of medicine	540 (0, 626)	951 (0, 386)	499 (0, 760)	356 (0, 444)	653 (0, 674)	630 (0, 674)	561 (0, 446)	< 0.001*
Blood transfusion	56 (0, 0)	76 (0, 0)	4 (0, 0)	19 (0, 0)	197 (0, 0)	85 (0, 0)	50 (0, 0)	< 0.001*
Consumable medical items for examination	75 (17, 61)	202 (41, 413)	54 (1, 42)	46 (13, 47)	122 (31, 113)	82 (17, 62)	117 (28, 105)	< 0.001*
Disposable medical materials for treatment	558 (60, 456)	1119 (111, 1838)	553 (142, 904)	337 (36, 246)	701 (118, 964)	667 (72, 458)	528 (42, 464)	< 0.001*
Disposable medical materials for surgery	20,810 (8150, 28728)	26,779 (3064, 39528)	23,675 (16005, 32591)	11,230 (751, 17825)	20,556 (2182, 26800)	24,046 (11919, 31436)	24,241 (12913, 30607)	< 0.001*
**Indirect Cost**								
Income loss	804 (0, 836)	1692 (288, 2691)	376 (0, 500)	577 (0, 454)	1249 (0, 1134)	925 (0, 989)	1119 (0, 1299)	< 0.001*
Travel expenses	134 (25, 202)	161 (51, 225)	122 (25, 180)	131 (22, 205)	186 (27, 358)	139 (26, 206)	127 (24, 192)	< 0.001*

Values are expressed as mean (lower quartile, upper quartile)

### Expenditure according to year

Enrolled patients were categorized by year to examine annual differences in the cost of sports injury surgery. Supplementary Table S3 documents the number of patients by statistical category for each year and finds that the percentage of patients by statistical category remains unchanged. Median total spending increased gradually over the first six years but has declined in the past two years. By year, the total cost of disposable medical materials for surgery, Surgical treatment, Laboratory examination, and Imaging account for the central part of the expenditure. The cost of disposable medical materials for examination, and the cost of disposable medical materials for treatment is declining yearly. In contrast, the cost of nursing service, laboratory examination and clinical diagnosis are increasing annually. For income loss, there is a significant decrease in 2019–2020 and 2021–2022 compared to 2015–2016 and 2017–2018.

**Table 2:** Expenditure of patients according to year category

Expenditure (¥)	Whole cohort (*n* = 8804)	2015–2016(*n* = 1379)	2017–2018(*n* = 2123)	2019–2020(*n* = 2348)	2021–2022(*n* = 2954)	*P* value
**Direct Cost**						
Total expenditure	38,691 (19680, 49640)	30,903 (11517, 42954)	38,336 (18441, 48544)	42,008 (23095, 53281)	39,946 (23624, 49304)	< 0.001*
General medical service	794 (300, 838)	753 (276, 821)	886 (375, 1050)	790 (354, 825)	751 (300, 750)	< 0.001*
General treatment operation	745 (195, 690)	789 (307, 835)	836 (290, 864)	774 (214, 614)	637 (152, 466)	< 0.001*
Nursing service	492 (158, 547)	376 (92, 371)	473 (158, 507)	531 (190, 587)	530 (164, 553)	< 0.001*
Pathological diagnosis	19 (0, 0)	12 (0, 0)	22 (0, 0)	21 (0, 0)	19 (0, 0)	0.794
Other	20 (0, 0)	84 (28, 112)	17 (0, 0)	0 (0, 0)	9 (0, 0)	< 0.001*
Laboratory examination	1363 (761, 1509)	1020 (604, 1093)	1031 (586, 1089)	1498 (845, 1801)	1654 (874, 1757)	< 0.001*
Imaging	1714 (682, 2361)	1524 (590, 2168)	2028 (931, 2644)	1748 (737, 2390)	1550 (551, 2099)	< 0.001*
Clinical diagnosis	123 (0, 90)	0 (0, 0)	27 (0, 0)	134 (0, 90)	241 (36, 100)	< 0.001*
Non-surgical treatment	401 (36, 342)	452 (47, 430)	486 (81, 434)	452 (49, 384)	275 (0, 153)	< 0.001*
Non-surgical treatment, including clinical physical therapy	186 (0, 184)	151 (0, 120)	199 (0, 208)	226 (0, 230)	160 (0, 138)	< 0.001*
Surgical treatment	5809 (2907, 8073)	3031 (1825, 3415)	4279 (2438, 5292)	7542 (3880, 10726)	6830 (3738, 8895)	< 0.001*
Surgical treatment, including anesthesia	901 (95, 1045)	127 (0, 228)	435 (200, 536)	1064 (0, 1175)	1467 (815, 1444)	< 0.001*
Surgical treatment, including surgical	4906 (2451, 7046)	2904 (1796, 3233)	3842 (2228, 4780)	6469 (3228, 9312)	5363 (2941, 7502)	< 0.001*
Rehabilitation	347 (30, 428)	206 (30, 126)	299 (31, 337)	503 (53, 687)	323 (20, 412)	< 0.001*
Total cost of medicine	540 (0, 626)	547 (0, 782)	554 (0, 695)	658 (0, 723)	434 (0, 404)	< 0.001*
Blood transfusion	56 (0, 0)	75 (0, 0)	57 (0, 0)	62 (0, 0)	41 (0, 0)	< 0.001*
Consumable medical items for examination	75 (17, 61)	118 (26, 93)	107 (26, 81)	57 (1, 43)	46 (17, 34)	< 0.001*
Disposable medical materials for treatment	558 (60, 456)	798 (186, 762)	721 (144, 592)	469 (63, 221)	400 (33, 214)	< 0.001*
Disposable medical materials for surgery	20,810 (8150, 28728)	14,935 (1121, 22999)	20,651 (6652, 28695)	22,487 (10408, 30700)	22,332 (11851, 29935)	< 0.001*
**Indirect Cost**						
Income loss	804 (0, 836)	1235 (0, 1460)	986 (0, 1190)	643 (0, 640)	601 (0, 570)	< 0.001*
Travel expenses	134 (25, 202)	141 (23, 222)	148 (26, 228)	152 (28, 225)	106 (23, 147)	< 0.001*

Values are expressed as mean (lower quartile, upper quartile)

### Expenditure according to the arthroscopy category

Because the diversity and complexity of sports injuries may result in varying hospitalization costs, subtypes of arthroscopic surgery, including the shoulder above, knee, ankle, and others, were analyzed. As shown in Table 3, the main components of total expenditure are similar according to statistical category and year. The median total expenditure for ankle arthroscopy was the lowest (36,917 yuan ≈ 5485 USD), and that for shoulder arthroscopy was the highest (51550 yuan ≈ 7660 USD).

**Table 3:** Expenditure of patients according to arthroscopy category

Expenditure (¥)	Whole cohort (*n* = 8804)	Other (*n* = 7147)	Shoulder Arthroscopy (*n* = 1241)	Knee arthroscopy (*n* = 304)	Ankle arthroscopy (*n* = 112)	*P* value
**Direct Cost**						
Total expenditure	38,691 (19680, 49640)	38,523 (16904, 50595)	51,550 (40703, 60028)	36,917 (27669, 42923)	34,177 (29194, 38209)	< 0.001*
General medical service	794 (300, 838)	864 (322, 920)	527 (300, 507)	484 (300, 525)	487 (279, 375)	< 0.001*
General treatment operation	745 (195, 690)	860 (230, 800)	267 (184, 254)	252 (148, 305)	205 (149, 199)	< 0.001*
Nursing service	492 (158, 547)	534 (160, 568)	505 (363, 583)	269 (147, 353)	273 (140, 330)	< 0.001*
Pathological diagnosis	19 (0, 0)	17 (0, 0)	9 (0, 0)	31 (0, 0)	10 (0, 0)	< 0.001*
Other	20 (0, 0)	24 (0, 0)	2 (0, 0)	6 (0, 0)	1 (0, 0)	< 0.001*
Laboratory examination	1363 (761, 1509)	1368 (710, 1489)	1821 (1230, 1939)	1215 (832, 1418)	1438 (1108, 1668)	< 0.001*
Imaging	1714 (682, 2361)	1763 (700, 2415)	1529 (1001, 1954)	1506 (540, 2122)	1411 (905, 1819)	< 0.001*
Clinical diagnosis	123 (0, 90)	133 (0, 81)	154 (90, 90)	61 (0, 90)	82 (72, 90)	< 0.001*
Non-surgical treatment	401 (36, 342)	436 (35, 355)	185 (107, 174)	276 (92, 336)	109 (61, 138)	< 0.001*
Non-surgical treatment, including clinical physical therapy	186 (0, 184)	190 (0, 184)	162 (92, 153)	175 (77, 199)	101 (46, 126)	< 0.001*
Surgical treatment	5809 (2907, 8073)	5289 (2599, 6848)	12,450 (11322, 13742)	7051 (5360, 8526)	7269 (6032, 7958)	< 0.001*
Surgical treatment, including anesthesia	901 (95, 1045)	825 (33, 1032)	3259 (3018, 3701)	757 (402, 952)	945 (815, 1033)	< 0.001*
Surgical treatment, including surgical	4906 (2451, 7046)	4460 (2245, 5797)	9191 (8163, 10208)	6295 (4614, 7639)	6324 (5155, 7035)	< 0.001*
Rehabilitation	347 (30, 428)	298 (23, 309)	693 (319, 1134)	535 (81, 872)	390 (20, 671)	< 0.001*
Total cost of medicine	160 (0, 0)	167 (0, 0)	207 (0, 444)	117 (0, 0)	77 (0, 79)	< 0.001*
Blood transfusion	56 (0, 0)	69 (0, 0)	1 (0, 0)	0 (0, 0)	0 (0, 0)	< 0.001*
Consumable medical items for examination	540 (0, 626)	595 (0, 676)	330 (0, 587)	318 (0, 508)	114 (0, 177)	< 0.001*
Disposable medical materials for treatment	558 (60, 456)	604 (58, 464)	663 (154, 901)	269 (60, 225)	559 (56, 314)	< 0.001*
Disposable medical materials for surgery	20,810 (8150, 28728)	20,366 (5991, 28763)	27,666 (17289, 35430)	21,814 (13072, 27736)	19,373 (16320, 22330)	< 0.001*
**Indirect Cost**						
Income loss	804 (0, 836)	926 (0, 1007)	333 (0, 465)	288 (0, 228)	75 (0, 0)	< 0.001*
Travel expenses	134 (25, 202)	134 (24, 204)	101 (23, 121)	144 (30, 204)	121 (25, 173)	< 0.001*

Values are expressed as mean (lower quartile, upper quartile)

### Expenditure according to age

Age at surgery significantly affects hospitalization costs, so we divided patients into five age groups. The highest total expenditure is in the > 65-year-old group (44,306 yuan ≈ 6583 USD), followed by the 46-65-year-old group (39,330 yuan ≈ 5844 USD), the 19-45-year-old group (37,736 yuan ≈ 5241 USD), and the 13-18-year-old group (37,368 yuan ≈ 5552 USD) and 1–12 years-old group (13671 yuan ≈ 2031 USD). Surgical treatment and the cost of disposable medical materials for surgery are the main parts of the total expenditure (Table 4).

**Table 4:** Expenditure of patients according to age category

Expenditure (¥)	Whole cohort (*n* = 8804)	1–12 years(*n* = 327)	13–18 years(*n* = 351)	19–45 years(*n* = 3463)	46–65 years(*n* = 2923)	> 65 years(*n* = 1740)	*P* value
**Direct Cost**							
Total expenditure	38,691 (19680, 49640)	13,671 (5182, 15899)	37,368 (18137, 52866)	37,736 (18132, 48302)	39,330 (21100, 48256)	44,306 (27807, 55588)	< 0.001*
General medical service	794 (300, 838)	426 (184, 505)	946 (300, 675)	697 (300, 695)	821 (340, 865)	950 (429, 1154)	< 0.001*
General treatment operation	745 (195, 690)	364 (88, 382)	500 (166, 467)	682 (184, 590)	771 (212, 690)	928 (246, 1015)	< 0.001*
Nursing service	492 (158, 547)	213 (72, 231)	358 (143, 469)	405 (142, 438)	512 (176, 552)	710 (239, 798)	< 0.001*
Pathological diagnosis	19 (0, 0)	18 (0, 0)	6 (0, 0)	15 (0, 0)	23 (0, 0)	23 (0, 0)	< 0.001*
Other	20 (0, 0)	15 (0, 0)	13 (0, 0)	17 (0, 0)	18 (0, 0)	26 (0, 0)	< 0.001*
Laboratory examination	1363 (761, 1509)	723 (415, 874)	1061 (591, 1263)	1183 (652, 1304)	1405 (840, 1528)	1854 (963, 2083)	< 0.001*
Imaging	1714 (682, 2361)	907 (405, 1175)	1563 (638, 2183)	1463 (540, 1992)	1776 (700, 2397)	2299 (1353, 2934)	< 0.001*
Clinical diagnosis	123 (0, 90)	61 (0, 36)	72 (0, 90)	99 (0, 90)	131 (0, 90)	190 (0, 90)	< 0.001*
Non-surgical treatment	401 (36, 342)	206 (0, 114)	245 (36, 290)	343 (36, 289)	402 (45, 363)	552 (37, 512)	< 0.001*
Non-surgical treatment, including clinical physical therapy	186 (0, 184)	52 (0, 0)	147 (0, 184)	166 (0, 168)	190 (0, 199)	230 (0, 204)	< 0.001*
Surgical treatment	5809 (2907, 8073)	3499 (1778, 4647)	6197 (3072, 9252)	5834 (2956, 8123)	6393 (3095, 8806)	5303 (2855, 6556)	< 0.001*
Surgical treatment, including anesthesia	901 (95, 1045)	690 (0, 781)	879 (234, 1054)	817 (191, 1032)	1064 (183, 1141)	884 (33, 1096)	< 0.001*
Surgical treatment, including surgical	4906 (2451, 7046)	2738 (1404, 3624)	5318 (2628, 7655)	5018 (2495, 7149)	5329 (2595, 7690)	4419 (2432, 5397)	< 0.001*
Rehabilitation	347 (30, 428)	94 (0, 91)	423 (30, 668)	359 (30, 490)	357 (30, 463)	329 (30, 390)	< 0.001*
Total cost of medicine	160 (0, 0)	57 (0, 0)	137 (0, 0)	145 (0, 0)	163 (0, 0)	220 (0, 0)	< 0.001*
Blood transfusion	56 (0, 0)	15 (0, 0)	44 (0, 0)	47 (0, 0)	39 (0, 0)	106 (0, 0)	< 0.001*
Consumable medical items for examination	540 (0, 626)	176 (0, 166)	430 (0, 493)	505 (0, 620)	595 (0, 657)	593 (0, 667)	< 0.001*
Disposable medical materials for treatment	558 (60, 456)	299 (33, 158)	427 (58, 327)	589 (56, 396)	554 (66, 509)	517 (69, 629)	< 0.001*
Disposable medical materials for surgery	20,810 (8150, 28728)	5364 (213, 6411)	21,790 (7651, 33096)	21,268 (6948, 29884)	20,337 (9145, 27167)	23,615 (14952, 30924)	< 0.001*
**Indirect Cost**							
Income loss	804 (0, 836)	96 (0, 0)	183 (0, 0)	861 (0, 786)	683 (0, 615)	1045 (0, 1459)	< 0.001*
Travel expenses	134 (25, 202)	99 (22, 141)	134 (25, 212)	148 (27, 222)	146 (26, 221)	91 (21, 103)	< 0.001*

Values are expressed as mean (lower quartile, upper quartile)

### Contributing factors to the total expenditure

Latent factors were entered into the GLM model as described above. Different age groups, genders, years of admission, ICF categories, surgical grades, occupation types, and arthroscopic surgery types were significant. Details on the GLM analysis are shown in Supplementary Table 2.

## Discussion

Sports injuries are expected not only in professional athletes but also in nationwide participation [11]. While the physical toll and long-term implications of such injuries are often discussed, an equally significant aspect that warrants attention is the financial burden associated with sports injuries’ surgeries. The cost of the sports recovery includes surgery, medication, rehabilitation, and other non-medical expenses, which was first studied in Southeast China. To prevent or minimize chronic disease prevalence, the Chinese government encouraged and developed sports promotion [12, 13]. The incidence of sports medicine increases when sports become prevalent in society. Understanding the financial implications of sports injuries’ surgeries is necessary for patients, healthcare professionals, and policymakers, as it can shed light on the accessibility and affordability of essential treatments. To our knowledge, this is the first comprehensive study of the economic burden of sports injuries, including different injury regions, periods, ages, and types of surgery in Southeast China. The cost considerations associated with sports injuries’ surgeries, examining factors contributing to the overall expenses and potential implications for athletes and the healthcare system.

Head and neck injuries in sports are a growing concern globally [14]. In the United States, there has been a significant increase in the number of sporting-related cervical fractures. Males have a higher incidence of neck injuries compared to females. The severity of sports injuries, including head and neck, also increases among high school athletes in the United States [15–17]. In Australia, different sports have varying patterns of head and neck injuries, with Australian rules football and rugby being notable for the frequency of such injuries [18, 19]. Both the United Kingdom and China face shared challenges in their sports industries, characterized by a lack of regulatory mechanisms and the enforcement of standardized protocols to address head and neck injuries, including concussions [20, 21]. The current organizational structures in both countries do not assume overall responsibility for establishing minimum standards or evaluating protocol implementation, resulting in avoidable brain injuries and potential long-term consequences for individuals. To address these issues, both countries must develop specific regulations and protocols prioritizing athlete safety and managing head and neck injuries effectively. This necessitates establishing or strengthening regulatory mechanisms, enforcing minimum standards, and rigorous evaluation of protocol efficacy to protect athletes and mitigate long-term impacts.

Analyzing the cost of sports injuries across different regions can provide valuable insights into the financial burden associated with specific injury types. The factors altered the total hospitalization expense divided into the complexity and severity, prevalence and incidence, diagnostic difficulties, rehabilitation and recovery, etc [17, 22, 23]. Not surprisingly, the head and neck injuries (s710) cost the highest expense due to their severity. Although sports injuries were rarely fatal, head and neck injuries could cause chronic disability, and the rehabilitation duration could be extensively long [24–26]. The total expense of shoulder injuries (s720) followed that of head and neck injuries. Shoulder injuries are commonly caused by contact sports, throwing sports, and overhead activities [23, 27, 28]. Additionally, identifying shoulder injuries could be complex and challenging. Multiple methods should be used in diagnosis, such as medical history, physical examination, X-rays, MRI scans, and ultrasound. The process requires not only the diagnostic equipment but also the experienced expertise. Shoulder injuries can encompass various conditions, including rotator cuff tears, labral tears, dislocations, and fractures. These injuries often require specialized diagnostic imaging, such as MRI scans, and complex surgical interventions, such as arthroscopic procedures or open surgeries. The complexity and severity of these injuries contribute to the high cost of treatment, involving expenses related to surgical procedures, hospital stays, post-operative care, and rehabilitation.

The tendency of the total expense of sports injuries in surgical procedures kept increasing. Although the trend is gradually increasing, it decreased from 2021 to 2022. This reduction can be attributed to the implementation of centralized procurement in Guangdong Province in 2022 [29], which has reduced the cost of orthopedic consumables. This measure has successfully lowered medical expenses by reducing potential costs for patients requiring surgery. The primary aim of this policy is to manage medical costs effectively and enhance resource utilization through the centralized procurement of orthopedic medical consumables by national organizations. Furthermore, manufacturers’ heightened market competition pressure is expected to improve product quality and service standards, ultimately benefiting patients. In recent years, the expectations of a return to play have changed in professional athletes and amateur sports enthusiasts [30]. Those patients require a short duration and complete recovery. Many anticipated that surgical management could bring them back to the same competitive intensity as before the injuries. This expectation shift has led to increased demand for advanced surgical procedures that aim to expedite recovery and optimize performance outcomes. For example, anterior cruciate ligament tear is one of the most common injuries in athletes. Different artificial grafts were invented and used clinically in anterior cruciate ligament reconstruction [31, 32]. Other innovative surgical techniques were reported for arthroscopy advancement [33–35]. Therefore, the cost of surgical fees and disposable items used in the operation kept increasing.

Arthroscopy is a minimally invasive approach for surgery in sports medicine. Since there is limited space and vision, the learning curve is long for orthopedic surgeons. However, studies reported that patients who underwent arthroscopy surgery had a shorter duration of rehabilitation. Arthroscopy surgery become popular in recent years in China. According to our findings, disposable items account for the highest percentage of the total expense. These disposable items are crucial in arthroscopic procedures, particularly ligament reconstruction or meniscal repair. The use of screws, allogenic ligaments, and artificial ligaments helps stabilize and support the injured joint, promoting optimal healing and functional recovery. Specialized equipment for suturing the meniscus enables precise and effective repair of this vital joint structure. While these disposable items contribute significantly to the success and outcomes of arthroscopic surgeries, they also contribute to the overall cost. As the demand for arthroscopy surgeries continues to rise in Southeast China, it becomes imperative to explore strategies to manage and optimize the utilization of these disposable items.

Regenerative ability fainted following aging, which is consistent with our results. The total expense increases when the age of the patients increases. As patients grow older, their bodies may have reduced capacity for natural healing and tissue regeneration. This can impact the outcomes of surgeries and necessitate additional resources or interventions, leading to increased expenses. The relationship between age and total surgery expense is essential for healthcare providers and policymakers. As the patient population ages, assessing and addressing the specific needs and challenges associated with older patients undergoing these procedures is crucial.

### Limitations

Although this study successfully established the economic costs associated with the analyzed phenomenon, it is essential to acknowledge the encountered limitations. Intangible costs refer to the non-monetary or non-financial impacts that are challenging to quantify or assign a specific value to. These costs may include pain, suffering, emotional distress, reduced quality of life, or societal implications beyond direct economic considerations. Future research endeavors should address this limitation by incorporating methods to evaluate and include intangible and economic costs.

## Conclusion

This study analyzed the expenditure on sports injury surgery. Significant differences were observed among the International Classification of Functioning categories, with head and neck injuries having the highest cost. Among the arthroscopy categories, shoulder arthroscopy had the highest median expenditure, while ankle arthroscopy had the lowest. Expenditure increased initially but declined in recent years. Medicine expenses decreased, while nursing expenses and surgical treatment fees increased annually. Age significantly influenced hospitalization costs, with the highest expenditure in the > 65-year-old group. Surgical treatment fees and the cost of disposable medical materials were the main components of total expenditure. These findings provide important insights into sports injury surgery expenditure patterns.

## Supplementary Information

Below is the link to the electronic supplementary material.

Supplementary Material 1

## Data Availability

No datasets were disclosed during the current study due to confidential privacy matter.

## References

[1] Wu A-M, Bisignano C, James SL, Abady GG, Abedi A, Abu-Gharbieh E, Alhassan RK, Alipour V, Arabloo J, Asaad M. Global, regional, and national burden of bone fractures in 204 countries and territories, 1990–2019: a systematic analysis from the global burden of disease study 2019. Lancet Healthy Longev. 2021;2(9):e580–92. 34723233 10.1016/S2666-7568(21)00172-0 PMC8547262

[2] Gribble PA, Bleakley CM, Caulfield BM, Docherty CL, Fourchet F, Fong DT-P, Hertel J, Hiller CE, Kaminski TW, McKeon PO. Evidence review for the 2016 international ankle consortium consensus statement on the prevalence, impact and long-term consequences of lateral ankle sprains. Br J Sports Med. 2016. 10.1136/bjsports-2016-096189 27259753

[3] Roos KG, Kerr ZY, Mauntel TC, Djoko A, Dompier TP, Wikstrom EA. The epidemiology of lateral ligament complex ankle sprains in National collegiate athletic association sports. Am J Sports Med. 2017;45(1):201–9. 27573356 10.1177/0363546516660980

[4] Carbone A, Rodeo S. Review of current Understanding of post-traumatic osteoarthritis resulting from sports injuries. J Orthop Res. 2017;35(3):397–405. 27306867 10.1002/jor.23341

[5] Valovich McLeod TC, Bay RC, Parsons JT, Sauers EL, Snyder AR. Recent injury and Health-Related quality of life in adolescent athletes. J Athl Train. 2009;44(6):603–10. 19911086 10.4085/1062-6050-44.6.603 PMC2775361

[6] Cohen David J, Reynolds Matthew R. Interpreting the results of Cost-Effectiveness studies. J Am Coll Cardiol. 2008;52(25):2119–26. 19095128 10.1016/j.jacc.2008.09.018 PMC2716087

[7] Bielska IA, Wang X, Lee R, Johnson AP. The health economics of ankle and foot sprains and fractures: A systematic review of English-language published papers. Part 2: the direct and indirect costs of injury. Foot. 2019;39:115–21. 29174064 10.1016/j.foot.2017.07.003

[8] Augustovski F, Briggs AH, Carswell C, Caulley L, Chaiyakunapruk N, Drummond M, Greenberg D, Husereau D, Loder E, Mauskopf J, et al. Consolidated health economic evaluation reporting standards 2022 (CHEERS 2022) statement: updated reporting guidance for health economic evaluations. Int J Technol Assess Health Care. 2022;38(1):e13. 35007499 10.1017/S0266462321001732

[9] Kohler F, Selb M, Escorpizo R, Kostanjsek N, Stucki G, Riberto M. Towards the joint use of ICD and ICF: A call for contribution. J Rehabil Med. 2012;44(10):805–10. 22990383 10.2340/16501977-1062

[10] Treuting R. Minimally invasive orthopedic surgery: arthroscopy. Ochsner J. 2000;2(3):158–63. 21765685 PMC3117522

[11] O’Dell MC, Jaramillo D, Bancroft L, Varich L, Logsdon G, Servaes S. Imaging of Sports-related injuries of the lower extremity in pediatric patients. Radiographics. 2016;36(6):1807–27. 27726754 10.1148/rg.2016160009

[12] Tu SJ, Jin C, Chen BT, Xu AY, Luo C, Wang XH. Study on the fusion of sports and medicine in China from 2012 to 2021: A bibliometric analysis via CiteSpace. Front Public Health. 2022;10:939557. 36187699 10.3389/fpubh.2022.939557 PMC9523407

[13] Xu J, Chen S. Sports and sports medicine in China in the post-Olympics era. Arthroscopy. 2010;26(4):506–7. 20362830 10.1016/j.arthro.2009.11.018

[14] Zhang W, Li H, Wang D, Xu G, Xu C, Li J, Zhang L, Tang P. The global research status and trends in ice and snow sports injuries from 1995 to 2022: a bibliometric and visualized analysis. Int J Environ Res Public Health 2023, 20(4). 10.3390/ijerph20042880 PMC9957478 36833576

[15] Pelletier JC. Sports related concussion and spinal injuries: the need for changing spearing rules at the National capital amateur football association (NCAFA). J Can Chiropr Assoc. 2006;50(3):195–208. 17549157 PMC1839959

[16] D’Lauro C, Jones ER, Swope LM, Anderson MN, Broglio S, Schmidt JD. Under-representation of female athletes in research informing influential concussion consensus and position statements: an evidence review and synthesis. Br J Sports Med. 2022. 10.1136/bjsports-2021-105045 35851519

[17] Sharma VK, Rango J, Connaughton AJ, Lombardo DJ, Sabesan VJ. The current state of head and neck injuries in extreme sports. Orthop J Sports Med. 2015;3(1):2325967114564358. 26535369 10.1177/2325967114564358 PMC4555583

[18] Yeomans C, Kenny IC, Cahalan R, Warrington GD, Harrison AJ, Hayes K, Lyons M, Campbell MJ, Comyns TM. The incidence of injury in amateur male rugby union: A systematic review and Meta-Analysis. Sports Med. 2018;48(4):837–48. 29299876 10.1007/s40279-017-0838-4 PMC5856893

[19] Health, AIo. Welfare: Sports injury in Australia. In. Canberra: AIHW. 2023.

[20] Fong DT, Chan YY, Mok KM, Yung PS, Chan KM. Understanding acute ankle ligamentous sprain injury in sports. Sports Med Arthrosc Rehabil Ther Technol. 2009;1:14. 19640309 10.1186/1758-2555-1-14 PMC2724472

[21] Li J, Liu J, Liu HW, Wei S, Jia YX, Li JJ. The trends in sports-related spinal cord injury in China. Spinal Cord. 2023;61(3):218–23. 36585484 10.1038/s41393-022-00872-0

[22] Liaghat B, Pedersen JR, Husted RS, Pedersen LL, Thorborg K, Juhl CB. Diagnosis, prevention and treatment of common shoulder injuries in sport: grading the evidence - a statement paper commissioned by the Danish society of sports physical therapy (DSSF). Br J Sports Med. 2023;57(7):408–16. 36261251 10.1136/bjsports-2022-105674 PMC10086287

[23] Lin DJ, Wong TT, Kazam JK. Shoulder injuries in the Overhead-Throwing athlete: Epidemiology, mechanisms of Injury, and imaging findings. Radiology. 2018;286(2):370–87. 29356641 10.1148/radiol.2017170481

[24] Usman S. Management of head and neck injuries by the sideline physician. Am Fam Physician. 2022;106(5):543–8. 36379500

[25] Veliz P, Ryan J, Eckner JT. Head, Neck, and traumatic brain injury among children involved in sports: results from the adolescent brain cognitive development study. J Adolesc Health. 2021;68(2):414–8. 32674966 10.1016/j.jadohealth.2020.06.004 PMC7855291

[26] Carmo GP, Grigioni J, Fernandes FAO, Alves de Sousa RJ. Biomechanics of traumatic head and neck injuries on women: a State-of-the-Art review and future directions. Biology (Basel) 2023, 12(1). 10.3390/biology12010083 PMC9855362 36671775

[27] FORTHOMME B, WIECZOREK V, CRIELAARD J-M FRISCHA. Shoulder pain among High-Level volleyball players and preseason features. Med Sci Sports Exerc. 2013;45(10):1852–60. 23575514 10.1249/MSS.0b013e318296128d

[28] Tooth C, Gofflot A, Schwartz C, Croisier JL, Beaudart C, Bruyère O, Forthomme B. Risk factors of overuse shoulder injuries in overhead athletes: A systematic review. Sports Health. 2020;12(5):478–87. 32758080 10.1177/1941738120931764 PMC7485028

[29] Province HSAoG: Healthcare Security Administration of Guangdong Province on the National Organization of Artificial Joints. Centralized banded purchasing and the use of notice. In: Healthcare Security Administration of Guangdong Province. 2022.

[30] Putukian M. Return to play: making the tough decisions. Phys Sportsmed. 1998;26(9):25–7. 20086849 10.3810/psm.1998.09.1125

[31] Xu C, Liu T, Wang M, Liu C, Li B, Lian Q, Chen T, Chen F, Qiao S, Wang Z. Comparison of proprioception recovery following anterior cruciate ligament reconstruction using an artificial graft versus an autograft. BMC Musculoskelet Disord. 2022;23(1):1056. 36463165 10.1186/s12891-022-06019-9 PMC9719127

[32] Jia ZY, Zhang C, Cao SQ, Xue CC, Liu TZ, Huang X, Xu WD. Comparison of artificial graft versus autograft in anterior cruciate ligament reconstruction: a meta-analysis. BMC Musculoskelet Disord. 2017;18(1):309. 28724372 10.1186/s12891-017-1672-4 PMC5517802

[33] Tian J, Mok TN, Sin TH, Zha Z, Zheng X, Teng Q, Hou H. Clinical outcomes of anterior tibiofibular ligament’s distal fascicle transfer versus ligament reconstruction with InternalBrace™ for chronic ankle instability patients. Arch Orthop Trauma Surg. 2022;142(10):2829–37. 34846587 10.1007/s00402-021-04214-2 PMC9474461

[34] Vega J, Poggio D, Heyrani N, Malagelada F, Guelfi M, Sarcon A, Dalmau-Pastor M. Arthroscopic all-inside atifl’s distal fascicle transfer for atfl’s superior fascicle reconstruction or biological augmentation of lateral ligament repair. Knee Surg Sports Traumatol Arthrosc. 2020;28(1):70–8. 30888451 10.1007/s00167-019-05460-z

[35] Zeng GL, Cai LM, Xie Q, Huang HB, Li YC, Su BY. Anterior talofibular ligament All-Inside arthroscopic reconstruction with InternalBrace™ for chronic lateral ankle instability. Med Sci Monit. 2022;28:e937699. 36199231 10.12659/MSM.937699 PMC9552571

