# Supplementary material for: Economic burden of sports injury in China: result from a single center of medical quality and safety monitor system
Source: Cost Eff Resour Alloc. 2025 Dec 2;24:3. doi: 10.1186/s12962-025-00659-z (PMC12777419; doi:10.1186/s12962-025-00659-z)
Supplement: Supplementary file 1 — Supplementary Material 1 [file 12962_2025_659_MOESM1_ESM.docx]

**Supplementary materials**

**Tsz-ngai MOK^1,2†^, Zhiguang HUANG^1†^, Huige HOU^2†^, Jing ZHAO^3^, Zihang CHEN^4^, Lek-Hang Cheang^5^, Man-seng Tam^6^, Tien-cheng YEH^2^, Dongyi FAN^2^, Jung CHEN^2^, Huajun WANG^2*^, Xiaofei ZHENG^2*^, Wai-kit MING^1*^**

^1^Department of Infectious Diseases and Public Health, City University of Hong Kong, Hong Kong SAR, China

^2^Department of Sports Medicine, The First Affiliated Hospital, Guangdong Provincial Key Laboratory of Speed Capability, The Guangzhou Key Laboratory of Precision Orthopedics and Regenerative Medicine, Jinan University, Guangzhou, China.

^3^State Key Laboratory of Quality Research in Chinese Medicine, Institute of Chinese Medical Sciences, Department of Pharmaceutical sciences, Faculty of Health Sciences, University of Macau, China

^4^Department of psychology, Li Ka Shing Faculty of Medicine, State Key Laboratory of Brain and Cognitive Sciences, The University of Hong Kong, Hong Kong SAR, China

^5^Department of Orthopedic Surgery, Centro Hospitalar Conde de Sao Januario, Macau, China

IAN WO Medical Center, Macao SAR, China

^6^Department of Orthopedic Surgery, Third Hospital of Shijiazhuang, HeBei, China

†These authors have contributed equally to this work and share first authorship

*Correspondence:

Huajun Wang, whj323@126.com

Xiaofei Zheng, zhengxiaofei12@163.com

Wai-kit MING, [wkming2@cityu.edu.hk](mailto:wkming2@cityu.edu.hk)

**Table of Contents**

[Supplementary Figure 1. Flowchart of this study 2](#_Toc537715517)

[Supplementary Table 1. CHEERS 2022 Checklist 2](#_Toc773507643)

[Supplementary Table 2. Detailed explanation of each column 5](#_Toc1624440610)

[Supplementary Table 3. Baseline characteristics of included patients 6](#_Toc1013727959)

[Supplementary Table 4. Results of generalized linear model analysis 9](#_Toc1738035262)


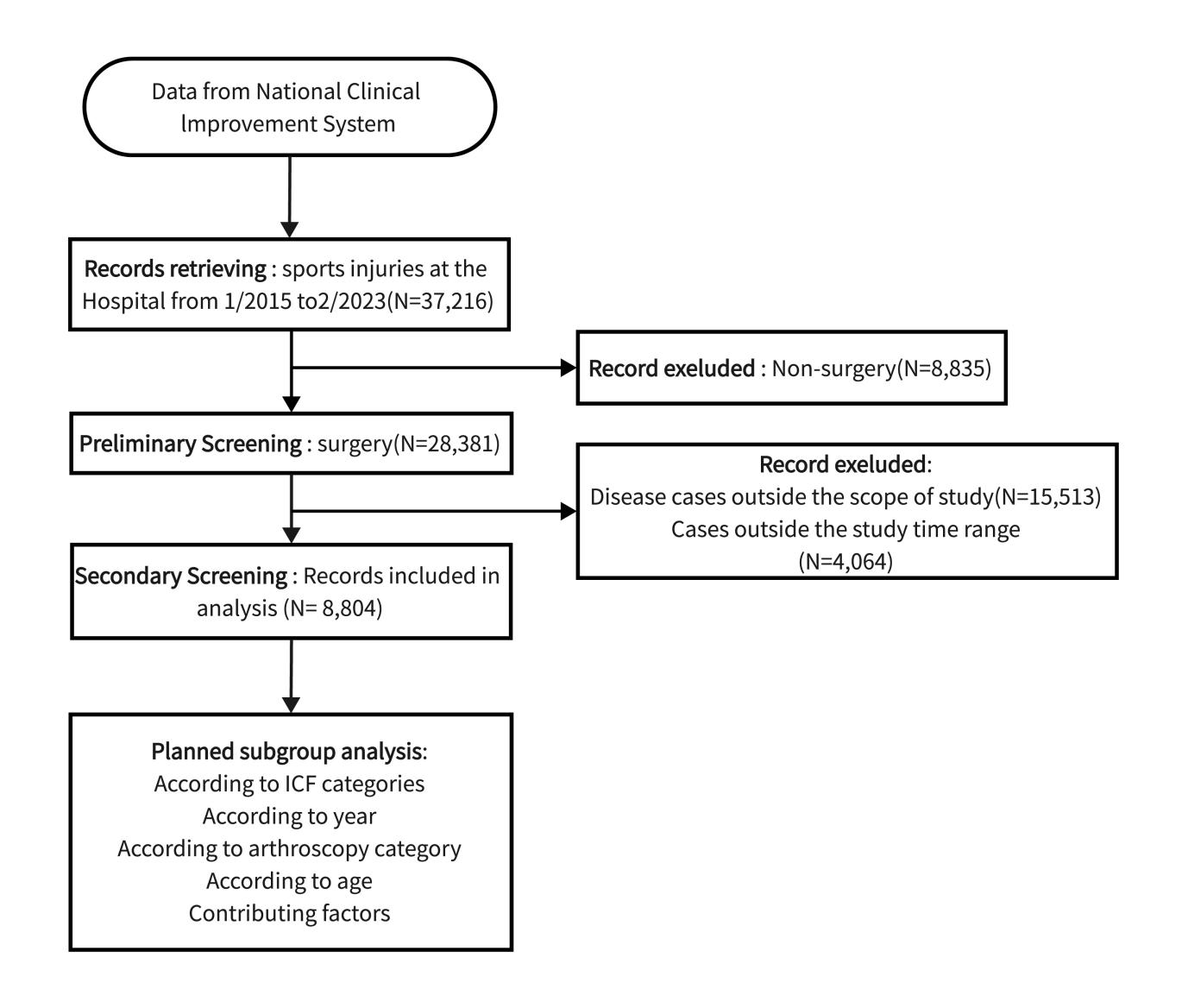


## Supplementary Figure 1. Flowchart of this study

Abbreviations: ICF: International Classification of Functioning, Disability, and Health

## Supplementary Table 1. CHEERS 2022 Checklist

|  | **Item** | **Guidance for Reporting** | **Reported in section** |
| --- | --- | --- | --- |
| **TITLE** | | |  |
| Title | 1 | Identify the study as an economic evaluation and specify the interventions being compared. | Title |
| **ABSTRACT** | | |  |
| Abstract | 2 | Provide a structured summary that highlights context, key methods, results and alternative analyses. | Abstract |
| **INTRODUCTION** | | |  |
| Background and objectives | 3 | Give the context for the study, the study question and its practical relevance for decision making in policy or practice. | Introduction, all paragraphs |
| **METHODS** | | |  |
| Health economic  analysis plan | 4 | Indicate whether a health economic analysis plan was developed and where available. | Method, all paragraphs, and Supplementary Figure 1 |
| Study population | 5 | Describe characteristics of the study population (such as age range, demographics, socioeconomic, or clinical characteristics). | Method, paragraph 3 |
| Setting and location | 6 | Provide relevant contextual information that may influence findings. | Discussion, paragraph 7 |
| Comparators | 7 | Describe the interventions or strategies being compared and why chosen. | Method, paragraph 4 |
| Perspective | 8 | State the perspective(s) adopted by the study and why chosen. | Method, paragraph 1 |
| Time horizon | 9 | State the time horizon for the study and why appropriate. | Method, paragraph 3 |
| Discount rate | 10 | Report the discount rate(s) and reason chosen. | Not applicable |
| Selection of outcomes | 11 | Describe what outcomes were used as the measure(s) of benefit(s) and harm(s). | Not applicable |
| Measurement of outcomes | 12 | Describe how outcomes used to capture benefit(s) and harm(s) were measured. | Not applicable |
| Valuation of outcomes | 13 | Describe the population and methods used to measure and value outcomes. | Method, paragraph 1-4 |
| Measurement and valuation of resources  and costs | 14 | Describe how costs were valued. | Method, paragraph 5-6 |
| Currency, price date, and conversion | 15 | Report the dates of the estimated resource quantities and unit costs, plus the currency and year of conversion. | Method, paragraph 5-6 |
| Rationale and  description of model | 16 | If modelling is used, describe in detail and why used. Report if the model is publicly available and where it can be accessed. | Method, paragraph 7 |
| Analytics and assumptions | 17 | Describe any methods for analysing or statistically transforming data, any extrapolation methods, and approaches for validating any model used. | Method, paragraph 7 |
| Characterizing heterogeneity | 18 | Describe any methods used for estimating how the results of the study vary for sub-groups. | Method section, paragraph 4 |
| Characterizing  distributional effects | 19 | Describe how impacts are distributed across different individuals or adjustments made to reflect priority populations. | Not applicable |
| Characterizing uncertainty | 20 | Describe methods to characterize any sources of uncertainty in the analysis. | Not reported |
| Approach to engagement with patients and others affected by the study | 21 | Describe any approaches to engage patients or service recipients, the general public, communities, or stakeholders (e.g., clinicians or payers) in the design of the study. | Not applicable |
| **RESULTS** | | |  |
| Study parameters | 22 | Report all analytic inputs (e.g., values, ranges, references) including uncertainty or distributional assumptions. | Method, paragraph 3 and results, all paragraphs |
| Summary of main results | 23 | Report the mean values for the main categories of costs and outcomes of interest and summarise them in the most appropriate overall measure. | Results, all paragraphs |
| Effect of uncertainty | 24 | Describe how uncertainty about analytic judgments, inputs, or projections  affect findings. Report the effect of choice of discount rate and time horizon, if applicable. | Discussion, paragraph 9 |
| Effect of engagement with patients and others affected by the study | 25 | Report on any difference patient/service recipient, general public, community, or stakeholder involvement made to the approach or findings of the study | Not applicable |
| **DISCUSSION** | | |  |
| Study findings, limitations, generalizability, and current knowledge | 26 | Report key findings, limitations, ethical or equity considerations not captured, and how these could impact patients, policy, or practice. | Discussion |
| **OTHER RELEVANT INFORMATION** | | | |
| Source of funding | 27 | Describe how the study was funded and any role of the funder in the identification, design, conduct, and reporting of the analysis | Abstract, paragraph 5 |
| Conflicts of interest | 28 | Report authors conflicts of interest according to journal or  International Committee of Medical Journal Editors requirements. | Declaration of interests |

Husereau D, Drummond M, Augustovski F, de Bekker-Grob E, Briggs AH, Carswell C, Caulley L, Chaiyakunapruk N, Greenberg D, Loder E, Mauskopf J, Mullins CD, Petrou S, Pwu RF, Staniszewska S; CHEERS 2022 ISPOR Good Research Practices Task Force. Consolidated Health Economic Evaluation Reporting Standards 2022 (CHEERS 2022) Statement: Updated Reporting Guidance for Health Economic Evaluations. BMJ. 2022;376:e067975.

The checklist is Open Access distributed in accordance with the terms of the Creative Commons Attribution (CC BY 4.0) license, which permits others to distribute, remix, adapt and build upon this work, for commercial use, provided the original work is properly cited. See: [http://creativecommons.org/licenses/by/4.0/.](http://creativecommons.org/licenses/by/4.0/)

## Supplementary Table 2. Detailed explanation of each column

| Columns | Explanation |
| --- | --- |
| General medical service | Fees incurred by doctors during diagnosis, including registration fees, consultation fees, etc. |
| General treatment operation | Doctors or nurses charge fees for general treatment operations, such as injection of drugs, infusion, removal of stitches, change of medication, and other routine medical operations. |
| Nursing service | The cost incurred by the medical care services provided by nursing staff. The nursing staff is responsible for the patient's daily care, including temperature measurement, blood pressure monitoring, condition observation, and assisting the patient in activities. Fees are billed according to the level of care and the duration of care. |
| Other | Other related fees that do not fall into the above fee categories. |
| Pathological diagnosis | Fees incurred by the hospital for pathological histologic examination of the patient are used to clarify the nature of the disease and the extent of the lesion. |
| Laboratory examination | Fees incurred by hospitals or clinics for various laboratory tests performed on patients to help doctors understand the patient's health condition. These tests include blood tests, urinalysis, etc. |
| Imaging | Fees incurred for various imaging tests (e.g., X-rays, CT, MRI, etc.) performed by the hospital to assist the doctor in making a diagnosis. |
| Clinical diagnosis | Doctors prescribe fees for clinical diagnostic programs, such as electrocardiograms, ultrasound examinations, etc., based on patients' symptoms and signs. |
| Non-surgical treatment | Fees incurred for treatment programs that are not performed through surgery. |
| Surgical treatment | Fees incurred for treatment items performed through surgery. |
| Rehabilitation | Fees incurred when the patient undergoes rehabilitation. |
| Total cost of medicine | Costs incurred for medications used by the patient. |
| Blood transfusion | Costs incurred for blood products given to the patient for transfusion as needed. |
| Travel expenses | Expenses incurred for transportation due to doctor's visits or home visits, etc. |
| Revenue deduction | This refers to a decrease in income due to unavoidable circumstances, such as a decrease in salary during sick leave. |
| Consumable medical items for examination | These expenses typically refer to the items consumed during medical examinations, such as disposable gloves, cotton swabs, medications, disinfectants, etc. |
| Disposable medical materials for treatment | These expenses refer to the disposable medical materials used during treatment, such as dressings, catheters, syringes, IV tubing, etc. |
| Disposable medical materials for surgery | These expenses refer to the disposable medical materials used during surgery, such as surgical drapes, scalpel blades, sutures, hemostatic agents, etc. |

## Supplementary Table 3. Baseline characteristics of included patients

| Variables | Whole cohort (n = 8804) | 2015-2016(n = 1379) | 2017-2018(n = 2123) | 2019-2020(n = 2348) | 2021-2022(n = 2954) |
| --- | --- | --- | --- | --- | --- |
| **Age** | 46.82 (20.49) | 45.72 (21.47) | 46.71 (20.61) | 46.75 (19.73) | 47.46 (20.51) |
| **gender** |  |  |  |  |  |
| male | 4992 (56.7%) | 808 (58.59%) | 1209 (56.95%) | 1317 (56.09%) | 1658 (56.13%) |
| female | 3812 (43.3%) | 571 (41.41%) | 914 (43.05%) | 1031 (43.91%) | 1296 (43.87%) |
| **ICF** |  |  |  |  |  |
| S710 | 57 (0.65%) | 7 (0.51%) | 12 (0.57%) | 18 (0.77%) | 20 (0.68%) |
| S720 | 987 (11.21%) | 28 (2.03%) | 224 (10.55%) | 364 (15.5%) | 371 (12.56%) |
| S730 | 2216 (25.17%) | 518 (37.56%) | 626 (29.49%) | 509 (21.68%) | 563 (19.06%) |
| S740 | 24 (0.27%) | 5 (0.36%) | 6 (0.28%) | 2 (0.09%) | 11 (0.37%) |
| S750 | 4496 (51.07%) | 596 (43.22%) | 1014 (47.76%) | 1269 (54.05%) | 1617 (54.74%) |
| S760 | 1024 (11.63%) | 225 (16.32%) | 241 (11.35%) | 186 (7.92%) | 372 (12.59%) |
| **level of surgery** |  |  |  |  |  |
| level One | 301 (3.42%) | 82 (5.95%) | 72 (3.39%) | 68 (2.9%) | 79 (2.67%) |
| level Two | 1242 (14.11%) | 378 (27.41%) | 290 (13.66%) | 270 (11.5%) | 304 (10.29%) |
| level Three | 5273 (59.89%) | 799 (57.94%) | 1334 (62.84%) | 1135 (48.34%) | 2005 (67.87%) |
| level Four | 1988 (22.58%) | 120 (8.7%) | 427 (20.11%) | 875 (37.27%) | 566 (19.16%) |
| **Profession** |  |  |  |  |  |
| Professional skill worker | 330 (3.75%) | 44 (3.19%) | 72 (3.39%) | 112 (4.77%) | 102 (3.45%) |
| Self-employed persons | 155 (1.76%) | 28 (2.03%) | 32 (1.51%) | 39 (1.66%) | 56 (1.9%) |
| civil servant | 120 (1.36%) | 14 (1.02%) | 24 (1.13%) | 32 (1.36%) | 50 (1.69%) |
| other | 4419 (50.19%) | 684 (49.6%) | 1000 (47.1%) | 1070 (45.57%) | 1665 (56.36%) |
| military | 7 (0.08%) | 2 (0.15%) | 2 (0.09%) | 2 (0.09%) | 1 (0.03%) |
| farmer | 476 (5.41%) | 58 (4.21%) | 128 (6.03%) | 132 (5.62%) | 158 (5.35%) |
| medical staff | 1 (0.01%) | 1 (0.07%) | 0 (0%) | 0 (0%) | 0 (0%) |
| student | 876 (9.95%) | 113 (8.19%) | 193 (9.09%) | 235 (10.01%) | 335 (11.34%) |
| education staff | 9 (0.1%) | 9 (0.65%) | 0 (0%) | 0 (0%) | 0 (0%) |
| Unemployed | 58 (0.66%) | 54 (3.92%) | 3 (0.14%) | 1 (0.04%) | 0 (0%) |
| service staff | 1 (0.01%) | 1 (0.07%) | 0 (0%) | 0 (0%) | 0 (0%) |
| manager | 23 (0.26%) | 2 (0.15%) | 8 (0.38%) | 5 (0.21%) | 8 (0.27%) |
| worker | 1086 (12.34%) | 190 (13.78%) | 395 (18.61%) | 385 (16.4%) | 116 (3.93%) |
| transport worker | 3 (0.03%) | 3 (0.22%) | 0 (0%) | 0 (0%) | 0 (0%) |
| retirees | 1240 (14.08%) | 176 (12.76%) | 266 (12.53%) | 335 (14.27%) | 463 (15.67%) |
| **medical insurance** | 0 (0%) | 0 (0%) | 0 (0%) | 0 (0%) | 0 (0%) |
| full public expense | 141 (1.6%) | 26 (1.89%) | 44 (2.07%) | 43 (1.83%) | 28 (0.95%) |
| Full self-pay | 5186 (58.91%) | 1115 (80.86%) | 1591 (74.94%) | 1340 (57.07%) | 1140 (38.59%) |
| other | 157 (1.78%) | 20 (1.45%) | 71 (3.34%) | 66 (2.81%) | 0 (0%) |
| other social insurance | 684 (7.77%) | 0 (0%) | 0 (0%) | 144 (6.13%) | 540 (18.28%) |
| Urban Residents Basic Medical Insurance | 1439 (16.34%) | 214 (15.52%) | 416 (19.59%) | 505 (21.51%) | 304 (10.29%) |
| Basic medical insurance for urban employees | 1196 (13.58%) | 3 (0.22%) | 1 (0.05%) | 250 (10.65%) | 942 (31.89%) |
| New rural cooperative medical care | 1 (0.01%) | 1 (0.07%) | 0 (0%) | 0 (0%) | 0 (0%) |
| **patient origin** |  |  |  |  |  |
| District where the hospital is located | 2999 (34.06%) | 544 (39.45%) | 678 (31.94%) | 630 (26.83%) | 1147 (38.83%) |
| The outer district (county) of the city where the hospital is located | 2182 (24.78%) | 247 (17.91%) | 470 (22.14%) | 596 (25.38%) | 869 (29.42%) |
| foreign country | 5 (0.06%) | 3 (0.22%) | 1 (0.05%) | 1 (0.04%) | 0 (0%) |
| Other provinces and cities | 4 (0.05%) | 0 (0%) | 1 (0.05%) | 1 (0.04%) | 2 (0.07%) |
| Other provinces (municipalities directly under the Central Government) | 1514 (17.2%) | 282 (20.45%) | 449 (21.15%) | 478 (20.36%) | 305 (10.32%) |
| Other districts and counties of this city | 15 (0.17%) | 0 (0%) | 2 (0.09%) | 1 (0.04%) | 12 (0.41%) |
| Other cities in this province | 2067 (23.48%) | 298 (21.61%) | 521 (24.54%) | 639 (27.21%) | 609 (20.62%) |
| Hong Kong, Macau, Taiwan | 18 (0.2%) | 5 (0.36%) | 1 (0.05%) | 2 (0.09%) | 10 (0.34%) |

Values are presented with Mean (SD) or counts (percentage).

## Supplementary Table 4. Results of generalized linear model analysis

| Variables | Estimate | standard error | 95% CI of estimate | Percent change | 95% CI of percent change | P |
| --- | --- | --- | --- | --- | --- | --- |
| Intercept | 9.561 | 0.0638 | 9.44 to 9.69 |  |  | <0.001∗ |
| Year(2021-2022 as reference) |  |  |  |  |  |  |
| 2015-2016 | -0.106 | 0.0217 | -0.15 to -0.06 | -10.06 | -13.88% to -6.23% | <0.001∗ |
| 2017-2018 | -0.015 | 0.0188 | -0.05 to 0.02 | -1.49 | -5.12% to 2.14% | 0.409 |
| 2019-2020 | 0.014 | 0.0189 | -0.02 to 0.05 | 1.41 | -2.35% to 5.17% | 0.452 |
| ICF category (s760 as reference) |  |  |  |  |  |  |
| s710(n = 57) | 0.275 | 0.0844 | 0.11 to 0.44 | 31.65 | 9.87% to 53.43% | 0.001* |
| s720(n = 987) | 0.071 | 0.0318 | 0.01 to 0.13 | 7.36 | 0.67% to 14.05% | 0.027* |
| s730(n = 2216) | -0.395 | 0.0255 | -0.45 to -0.35 | -32.63 | -36% to -29.26% | <0.001∗ |
| s740(n = 24) | -0.006 | 0.128 | -0.26 to 0.25 | -0.60 | -25.54% to 24.34% | 0.962 |
| s750(n = 4496) | 0.194 | 0.0239 | 0.15 to 0.24 | 21.41 | 15.72% to 27.1% | <0.001∗ |
| Sex (Women as reference) | 0.115 | 0.0147 | 0.09 to 0.14 | 12.19 | 8.96% to 15.42% | <0.001∗ |
| Surgical grading(level Four as reference) |  |  |  |  |  |  |
| level One | 0 | 0.0404 | -0.08 to 0.08 | 0.00 | -7.92% to 7.92% | 0.991 |
| level Two | 0.621 | 0.0373 | 0.55 to 0.69 | 86.08 | 72.47% to 99.68% | <0.001∗ |
| level Three | 0.813 | 0.041 | 0.73 to 0.89 | 125.47 | 107.35% to 143.58% | <0.001∗ |
| Occupation category(Professional skill worker as reference) |  |  |  |  |  |  |
| service | 1.583 | 0.6197 | 0.37 to 2.8 | 386.95 | -204.51% to 978.41% | 0.011* |
| Self-employed | 0.274 | 0.0603 | 0.16 to 0.39 | 31.52 | 15.98% to 47.07% | <0.001∗ |
| civil servant | 0.214 | 0.066 | 0.08 to 0.34 | 23.86 | 7.84% to 39.89% | 0.001* |
| manager | 0.105 | 0.1335 | -0.16 to 0.37 | 11.07 | -17.99% to 40.13% | 0.431 |
| education staff | -0.004 | 0.2099 | -0.42 to 0.41 | -0.40 | -41.38% to 40.58% | 0.985 |
| military | 1.117 | 0.2368 | 0.65 to 1.58 | 205.57 | 63.74% to 347.39% | <0.001∗ |
| farmer | 0.258 | 0.0454 | 0.17 to 0.35 | 29.43 | 17.92% to 40.95% | <0.001∗ |
| other | 0.104 | 0.0355 | 0.03 to 0.17 | 10.96 | 3.24% to 18.68% | 0.004* |
| retirees | 0.121 | 0.0409 | 0.04 to 0.2 | 12.86 | 3.81% to 21.91% | 0.003* |
| Unemployed | 0.289 | 0.0902 | 0.11 to 0.47 | 33.51 | 9.91% to 57.11% | 0.001* |
| student | 0.088 | 0.0461 | 0 to 0.18 | 9.20 | -0.67% to 19.07% | 0.058 |
| medical staff | 0.177 | 0.6199 | -1.04 to 1.39 | 19.36 | -125.66% to 164.39% | 0.776 |
| transport worker | -0.828 | 0.3593 | -1.53 to -0.12 | -56.31 | -87.08% to -25.54% | 0.021* |
| worker | 0.119 | 0.0392 | 0.04 to 0.2 | 12.64 | 3.98% to 21.29% | 0.002* |
| arthroscopy category(Ankle arthroscopy as reference) |  |  |  |  |  |  |
| other(n = 7147) | 0.097 | 0.0624 | -0.03 to 0.22 | 10.19 | -3.29% to 23.66% | 0.12 |
| Shoulder Arthroscopy(n = 1241) | 0.364 | 0.0487 | 0.27 to 0.46 | 43.91 | 30.17% to 57.64% | <0.001∗ |
| Knee arthroscopy(n = 304) | 0.384 | 0.0232 | 0.34 to 0.43 | 46.81 | 40.14% to 53.49% | <0.001∗ |
| Age(>65 years as reference) |  |  |  |  |  |  |
| 1-12 years | -0.872 | 0.0478 | -0.97 to -0.78 | -58.19 | -62.11% to -54.27% | <0.001∗ |
| 13-18 years | -0.157 | 0.0473 | -0.25 to -0.07 | -14.53 | -22.45% to -6.61% | 0.001* |
| 19-45 years | -0.151 | 0.0234 | -0.2 to -0.11 | -14.02 | -17.96% to -10.07% | <0.001∗ |
| 46-65 years | -0.11 | 0.021 | -0.15 to -0.07 | -10.42 | -14.1% to -6.73% | <0.001∗ |

*indicates statistical significance.

Abbreviations: CI, confidence interval; ICF:International Classification of Functioning, Disability, and Health
